# Characterization of Extrachromosomal Circular DNA in Primary and Cisplatin-Resistant High-Grade Serous Ovarian Cancer

**DOI:** 10.3390/genes16050517

**Published:** 2025-04-29

**Authors:** Youya Wang, He Li, Qinglan Li, Yi Li, Hao Wu, Yan Ge, Xingnuo Zhu, Zhiguo Zheng, Zhongsheng Sun

**Affiliations:** 1 Institute of Genomic Medicine, Wenzhou Medical University, Wenzhou 325000, China; youya_wang@163.com (Y.W.); lihe2944964125@gmail.com (H.L.); wu_hao2021@163.com (H.W.); gy1978185574@163.com (Y.G.); believe_my_ship@163.com (X.Z.); 2 Institute of Zoology, Chinese Academy of Sciences, Beijing 100101, China; liqinglan@biols.ac.cn (Q.L.); liyieebio@zju.edu.cn (Y.L.); 3 College of Life Sciences, University of the Chinese Academy of Sciences, Beijing 100049, China; 4 Hangzhou Institute of Medicine (HIM), Chinese Academy of Sciences, Hangzhou 310022, China; 5 Zhejiang Cancer Hospital, Hangzhou 310022, China

**Keywords:** HGSOC, cisplatin resistance, circle-Seq, eccDNA

## Abstract

Background: Cisplatin resistance is a major cause of tumor recurrence and mortality in high-grade serous ovarian cancer (HGSOC). Extrachromosomal circular DNA (eccDNA) has emerged as a critical factor in tumor evolution and drug resistance. However, the specific contribution of eccDNA to cisplatin resistance in HGSOC remains unclear. Methods: We performed whole-genome sequencing, Circle-Seq, and RNA-Seq in four pairs of primary and cisplatin-resistant (cisR) HGSOC cell lines to characterize genome-wide eccDNA distribution and features. Functional enrichment analyses were subsequently conducted on differentially expressed eccDNA-related genes. Results: In the SKOV3 cisR cell line, we identified a large extrachromosomal circular DNA (ecDNA) carrying the *HIF1A* gene, which regulates DNA repair, drug efflux, and epithelial–mesenchymal transition, contributing to cisplatin resistance. Using Circle-Seq, we detected a total of 161,062 eccDNAs, most of which were less than 1000 bp and distributed across all chromosomes. Notably, the number of eccDNAs on chromosome 21 differed significantly between the primary and cisR cell lines. Additionally, eccDNAs were predominantly located in non-coding repetitive elements. Functional analysis of eccDNA-related differentially expressed genes revealed that, compared to primary cell lines, cisR cell lines were associated with mitotic spindle assembly, regulation of vascular permeability, and cell differentiation. eccDNA-related genes involved in these pathways include *MISP*, *WIPF1*, *RHOD*, *KRT80*, and *PLVAP*. Conclusions: Our findings suggest that eccDNAs, particularly ecDNA amplifications like *HIF1A*, contribute significantly to cisplatin resistance mechanisms in HGSOC. These insights highlight eccDNA as a potential target for overcoming therapeutic resistance and improving treatment outcomes in ovarian cancer.

## 1. Introduction

Ovarian cancer is the most lethal gynecological malignancy, accounting for approximately 5% of all cancer-related deaths among women [1]. High-grade serous ovarian cancer (HGSOC) is the most common histological subtype. Approximately 70–80% of patients with HGSOC are diagnosed at an advanced stage with widespread metastasis, resulting in a dismal five-year overall survival rate of less than 30% [2]. The current standard-of-care therapy involves debulking surgery followed by combinatorial chemotherapy with taxane and platinum agents [2]. To date, cisplatin remains a cornerstone first-line chemotherapeutic drug for ovarian cancer treatment [3]. Although the majority of patients initially exhibit sensitivity to cisplatin-based treatment, over 70% experience cycles of disease relapse and retreatment, eventually developing progressive, chemoresistant tumors that lead to treatment-resistant disease and poor outcomes [4]. Therefore, comprehensive characterization of genomic alterations and gene expression patterns associated with cisplatin resistance in ovarian cancer is critical for elucidating its molecular mechanisms and identifying novel therapeutic targets with potential clinical utility.

Extrachromosomal circular DNA is a non-mitochondrial, double-stranded circular DNA molecule derived from chromosomal DNA, with diverse sizes and functions [5,6]. Extrachromosomal circular DNA can generally be classified into two distinct categories based its size and function: (1) Small extrachromosomal circular DNA (eccDNA, <10 kb) that carries only small DNA fragments and may have diverse regulatory functions; and (2) large extrachromosomal DNA (ecDNA, >10kb) that is cancer-specific extrachromosomal circular DNA and can carry multiple intact genes and regulatory elements, contributing to its high copy number amplification [7]. Due to significant structural and functional differences, ecDNA and eccDNA are typically studied independently. Large ecDNAs, initially referred to as double minutes (DMs), are widely present in various tumor types and commonly drive oncogene amplification. Due to the lack of centromeres, ecDNA segregates randomly into daughter cells during mitosis, promoting intratumoral genetic heterogeneity and tumor evolution. Mischel et al. demonstrated in glioblastoma that ecDNA carrying the mutant of *EGFR* (*EGFRvIII*) can induce resistance to *EGFR* inhibitors through changes in its own copy number [8]. However, the precise role of ecDNA in cisplatin resistance in ovarian cancer remains unexplored. In contrast, small eccDNA is ubiquitously found in both normal and cancerous cells, typically encompassing genetic elements such as short non-coding sequences, transposable elements, microsatellites, or protein-coding fragments [6]. Therefore, these small eccDNAs participate in diverse biological processes, including activating endogenous immune responses [9,10] and serving as templates for regulatory RNAs or genes [11,12].

Both ecDNA and eccDNA are results of genomic instability. HGSOC is characterized by a ubiquitous *TP53* mutation, and in about half of HGSOC cases, there is evidence of mutations of homologous recombination repair. Therefore, it is reasonable to hypothesize that extrachromosomal circular DNA plays a crucial role in cisplatin resistance. In this study, we performed a systematic multi-omics characterization of extrachromosomal circular DNAs (ecDNA/eccDNA) across four pairs of matched primary and cisplatin-resistant ovarian cancer cell lines. By integrating whole-genome sequencing, Circle-Seq, and transcriptomic data, we explored the potential mechanistic roles of extrachromosomal circular DNAs in cisplatin resistance, aiming to identify novel therapeutic targets to improve outcomes for ovarian cancer patients.

## 2. Materials and Methods

### 2.1. Cell Lines and Cell Culture

We obtained four matched pairs of human ovarian cancer primary and cisR cell lines from Zhejiang Cancer Hospital: OVCAR4 and OVCAR4-cisR, KURAMOCHI and KURAMOCHI-cisR, A2780 and A2780-cisR, and SKOV3 and SKOV3-cisR. Cells were cultured in DMEM/F-12 (1:1) medium containing 10% fetal bovine serum (FBS) under standard conditions (37 °C, 5% CO_2_, humidified atmosphere). Adherent monolayers were passaged at 90% confluence using 0.25% trypsin-EDTA. All cell lines tested negative for mycoplasma contamination.

### 2.2. Cell Counting Kit-8 Assay

Cell viability was assessed using the CCK-8 assay (YEASEN, Cat. 40203ES76), following the manufacturer’s instructions. Cells were seeded in 96-well plates at a density of 5000 cells per well and treated with cisplatin at varying concentrations. After incubation at 37 °C with 5% CO_2_, 10 μL CCK-8 solution was added to each well at 24, 48, 72, 96, and 120 h post-treatment. Following a 3-h incubation, absorbance was measured at 450 nm using a microplate reader.

### 2.3. Whole-Genome Sequencing, Data Preprocessing, and Alignment

Genomic DNA was extracted from four pairs of primary and cisplatin-resistant ovarian cancer cell lines. Sequencing libraries were prepared by BGI-Hangzhou and sequenced on the DNBSEQ-T7 platform (PE 150 mode), achieving an average coverage depth of 100X per sample. Low-quality bases and adapter sequences were removed prior to alignment. Cleaned reads were mapped to the human reference genome (GRCh38) using BWA-MEM v0.7.17 [13], and filtered for PCR duplicates via Samblaster v0.1.26 [14]. Post-alignment processing included sorting and indexing BAM files with Samtools, followed by base-quality score recalibration and indel region realignment using GATK v4.3.0.0 [15].

### 2.4. Somatic Mutation Calling, Filtering, and Annotation

SNV/INDELs and short indels were identified using Strelka2 [16] and MuTect2 [17]. For all analyses of WGS, we used data from unmatched lymphoblastoid cell lines [18] as controls, processed using the same pipeline as the tumor cell lines. Mutations detected by both tools were merged and annotated using ANNOVAR v2020.06.07 [19].

### 2.5. Mutational Signature Analysis of Single-Nucleotide Substitutions

First, we utilized the Python tool SigProfilerMatrixGenerator v1.2 to construct a 96-channel mutation count matrix. Next, SigProfilerExtractor v1.1.23 [20] was employed to extract de novo mutational signatures, with parameters set as follows: --min_sig 3, --max_sig 10, and --max_iter 500. Finally, we used SigProfilerAssignment v0.0.32 to decompose the identified de novo mutational signatures into established COSMIC single-base substitution signatures (https://cancer.sanger.ac.uk/signatures/sbs/, accessed on 12 November 2023).

### 2.6. Somatic Structural Variant Calling and Filtering

Somatic Structural Variants (SVs) were called using SvABA v1.1.0 [21], Manta v1.6.0 [22], and Delly v1.1.6 [23] with default parameters. SVs identified by at least two independent algorithms were considered high-confidence SVs and retained for downstream analysis.

### 2.7. Copy Number Alterations Analysis

Copy number alterations were inferred using FACETS v0.6.1 [24] with default parameters, which performs allele-specific segmentation and adjusts copy-number estimates based on tumor purity and ploidy. The resulting segmented log-ratio and allele frequency data were converted to GISTIC2-compatible format and analyzed with GISTIC2 [25] to identify significantly recurrent CNA events.

### 2.8. Detection of Complex Rearrangements

CNVKit v0.9.8 [26] was utilized to detect genomic segments exhibiting copy number alterations. Amplified genomic regions exceeding 50 kb with copy numbers greater than 4.3 were selected and subsequently reconstructed using AmpliconArchitect v1.2.17 [27]. These amplified segments were further classified into distinct categories including ecDNA, BFB, linear amplifications, and complex non-cyclic amplification using AmpliconClassifier v0.4.13. Additionally, junction-balanced genome graphs were generated with JaBbA v1.1 [28], whose outputs were analyzed using gGnome v0.1 [28] to infer complex SV types.

### 2.9. Circular DNA Sequencing

Total DNA from paired primary and cisR cell lines was enriched for circular DNA. Mitochondrial circular DNA and linear DNA were removed before performing rolling-circle amplification and library preparation. Linear DNA was digested with an exonuclease master mix (Epicentre Biotechnologies, Madison, WI, USA), while mitochondrial DNA was cleaved using PacI endonuclease (New England Biolabs, Ipswich, MA, USA). Circular DNA was purified with AMPure XP beads (Beckman Coulter, Brea, CA, USA), amplified via Phi29 polymerase (REPLI-g Midi Kit, Qiagen, Hilden, Germany), and sonicated for fragmentation. Libraries were prepared with YEASEN kits and sequenced on the Illumina NovaSeq 6000 platform (PE 150 bp). eccDNA was identified with Circle-Map++ (modified from Circle-Map v1.1.4) under stringent criteria: split reads ≥ 2, circle score ≥ 200, coverage continuity ≤ 0.1, start/end coverage increase ≥ 0.33, and coverage standard deviation below the mean.

### 2.10. Identification of Unique and Shared eccDNAs

To identify unique and shared eccDNA, eccDNAs from the same chromosome were considered shared if their upstream and downstream coordinates were within ±50 bp; otherwise, they were classified as unique eccDNAs.

### 2.11. Identification of Unique Circular DNA Genes

Unique genes in primary ovarian cancer were identified as those present in at least three primary ovarian cancer cell lines but absent in cisR ovarian cancer cell lines. Conversely, unique genes in cisR ovarian cancer were defined as those present in at least three cisR ovarian cancer cell lines but absent in primary ovarian cancer cell lines.

### 2.12. RNA Sequencing Analysis

Total RNA from four paired cell lines (three biological replicates per line) was sequenced on the DNBSEQ-T7 platform (BGI-Hangzhou (Hangzhou, China), PE 150 mode). Raw reads were trimmed with TrimGalore and aligned to GRCh38 using HISAT2 v2.2.1 [29]. Gene expression quantification with FeatureCounts v2.0.6 [30] was followed by differential expression analysis via DESeq2 [31]. Functional enrichment of differentially expressed genes was performed for GO terms and KEGG pathways.

### 2.13. Statistical Analysis

All statistical analyses were conducted in R v4.3.1. Group comparisons utilized two-tailed paired Student’s *t*-tests, while correlations were assessed via Spearman’s method. Significance thresholds were set as * *p* < 0.05, ** *p* < 0.01, *** *p* < 0.001 and **** *p* < 0.0001.

## 3. Results

### 3.1. Establishment of Primary and Cisplatin-Resistant HGSOC Cell Lines

**Figure 1:**
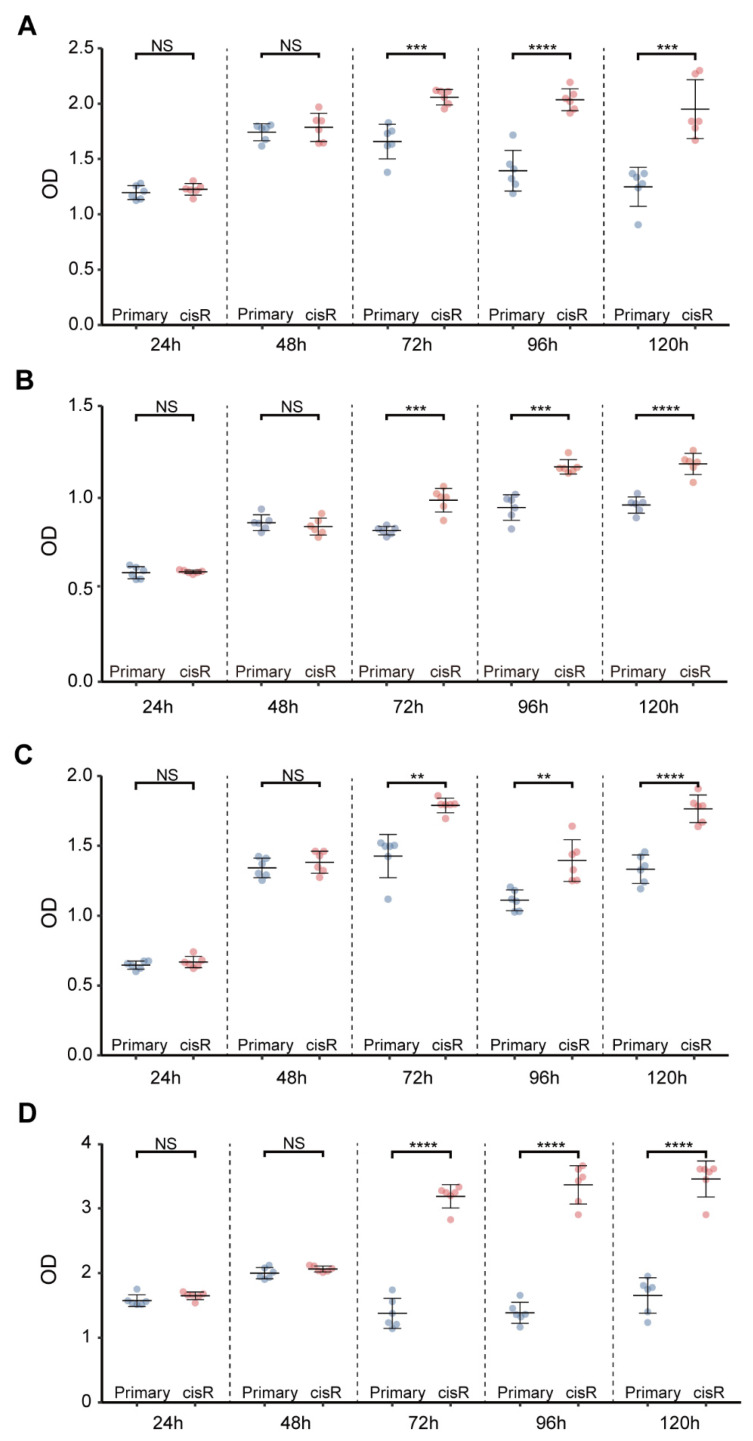
Cisplatin resistance in ovarian cancer cell lines. (**A**–**D**) Cisplatin resistance analysis of OVCAR4-cisR, KURAMOCHI-cisR, A2780-cisR and SKOV3-cisR from top to bottom, respectively (Paired *t*-test). Statistical significance is indicated as follows: NS = not significant, ** *p* < 0.01, *** *p* < 0.001, **** *p* < 0.0001.

In this study, we obtained four matched pairs of primary and cisR human ovarian cancer cell lines. Detailed descriptions and classifications of these cell lines are provided in Supplementary Table S1. A2780 and SKOV3 are frequently used in ovarian cancer research, particularly in studies of HGSOC [32]. While OVCAR4 and KURAMOCHI are less commonly used in HGSOC research, their genomic profiles exhibit remarkable similarities to those of HGSOC, making them valuable models for studying HGSOC [32]. Prior to sequencing, we validated the cisplatin-resistant phenotypes in four pairs of ovarian cancer cell lines. Following a 72 h cisplatin treatment, we observed significant differences in cell viability between each matched primary and cisR cell line pair (Figure 1), confirming the successful establishment of cisplatin-resistant phenotypes. Subsequently, to elucidate the genomic landscape and molecular characteristics associated with extrachromosomal circular DNA, we performed comprehensive genomic and extrachromosomal circular DNA profiling on these ovarian cancer cell line pairs. Transcriptomic analyses were integrated to generate detailed differential expression profiles of extrachromosomal circular DNA-related genes, thereby uncovering their potential contributions to cisplatin resistance mechanisms in HGSOC cell lines.

### 3.2. Genomic Features and Mutational Signatures in Primary and Cisplatin-Resistant HGSOC Cell Lines

**Figure 2:**
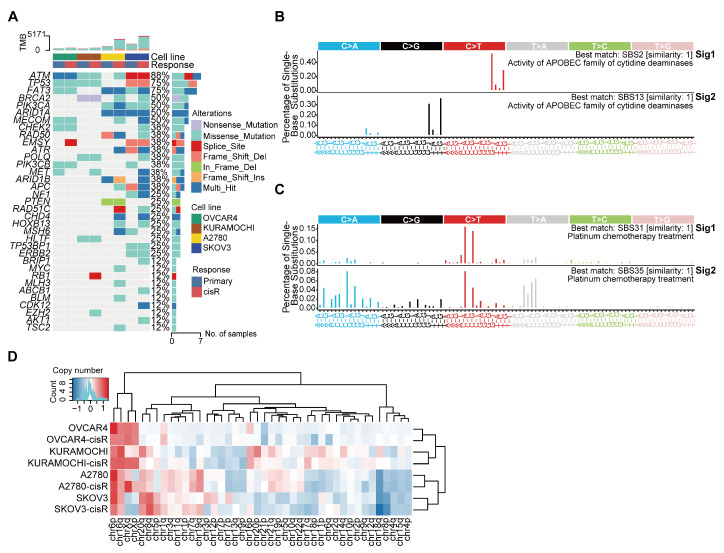
Genomic characterizations of HGSOC cell lines. (**A**) Overview of somatic alterations in driver genes. (**B**) Mutation signatures identified in primary cell lines. (**C**) Mutation signatures identified in cisR cell lines. (**D**) Copy number profiles for all cell lines.

We first characterized the genomic differences between primary and cisR cell lines using whole-genome sequencing. Compared to primary cell lines, cisR cell lines exhibited higher genomic instability and tumor mutation burden. Consistent with previous studies [33], frequently mutated genes in both primary and cisR cell lines included *TP53*, *ATM*, *BRCA1*, and *BRCA2* (Figure 2A; Supplementary Table S2). To investigate whether the mutational difference may be attributed to differential environmental activity or endogenous mutational processes, we quantitatively and comparatively assessed the activities of all operative mutational signatures. We identified four distinct single-base substitution (SBS) signatures. SBS31 and SBS35, which are specifically associated with platinum-based chemotherapy, were uniquely enriched in cisR cell lines (Figure 2C). Conversely, SBS signatures identified in primary cell lines included SBS2 and SBS13, signatures linked to activity of APOBEC enzymes, previously shown to confer cisplatin sensitivity through induction of DNA damage [34,35] (Figure 2B). These observations highlight that cisplatin exposure significantly reshapes mutational signatures, suggesting specific mutational processes drive cisplatin resistance. Chromosomal copy number alterations (CNAs) are a frequent genetic event observed in HGSOC. To identify CNA patterns potentially associated with cisplatin resistance, we systematically compared genome-wide copy-number profiles across four paired primary and cisR cell lines. Frequent copy number gains were identified on chromosomes 6p, 8q, 16q, 20q, Xp, and Xq, while copy-number losses occurred on chromosomes 4p, 4q, 8p, 15q, and 18q. However, these CNAs did not significantly differ between paired primary and cisR cell lines, suggesting these alterations represent general genomic characteristics of HGSOC rather than specific drivers of acquired cisplatin resistance (Figure 2D).

### 3.3. Characteristics of Large ecDNA in Primary and cisR HGSOC Cell Lines

**Figure 3:**
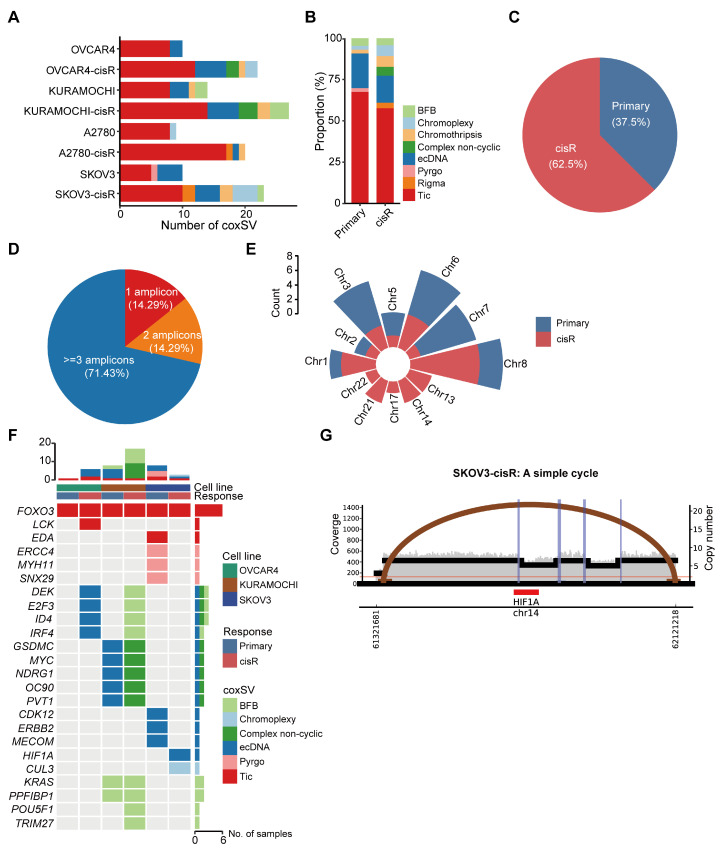
Distribution of complex structural variations and ecDNAs in HGSOC cell lines. (**A**) Numbers of complex structural variations and ecDNAs identified in primary and cisR cell lines. coxSVs were classified as Tic, Rigma, Pyrgo, ecDNA, complex non-cyclic, chromothripsis, chromoplexy and BFB. (**B**) Relative frequency (as a percentage) of coxSVs in primary and cisR cell lines. (**C**) The percentage of ecDNA in primary and cisR cell lines. (**D**) Distribution of amplicons constituting ecDNA. (**E**) Distribution number of ecDNAs in each chromosome. (**F**) Oncogenes annotated by coxSVs and ecDNAs. (**G**) Structure of ecDNA carrying *HIF1A* in SKOV3-cisR cell line.

Complex structural variants (coxSVs) refer to multiple (≥3) DNA junctions in distinct topologies within the reference genome that yield one or more copies of complex rearranged alleles [28]. For example, chromothripsis is a phenomenon characterized by a single catastrophic event resulting in numerous somatic genome rearrangements and has been observed across various tumor types [36]. Recent studies have suggested that ecDNA may arise from the interplay between mutations and complex structural variants [37]. These structural variants can fragment chromosomes and expose DNA ends to error-prone repair processes, such as non-homologous end joining (NHEJ) or alternative end joining (alt-EJ), which may mediate circularization of genomic segments. In particular, chromothripsis-induced DNA fragmentation can create multiple discrete fragments that re-ligate into ecDNA under selective pressure. To investigate these processes, we utilized JaBbA v1.1 and AmpliconArchitect v1.2.17 software to quantify and characterize the differences in coxSVs and ecDNAs between primary and cisR cell lines. cisR cell lines exhibited a significantly higher abundance of coxSVs and ecDNAs compared to primary cell lines. Specifically, we identified a total of 201 coxSVs and 15 ecDNAs in cisR cell lines, compared with 84 coxSVs and 9 ecDNAs in primary cell lines (Figure 3A,C; Supplementary Tables S3 and S4). Moreover, six chromothripsis and five complex non-cyclic structural variants were exclusively detected in cisR cells, suggesting that catastrophic genomic rearrangements may provide substrates necessary for ecDNA formation (Figure 3B). Interestingly, most ecDNAs are composed of multiple amplicons and are not formed by the circularization of a single amplified fragment, indicating their complex genomic origin (Figure 3D). Furthermore, ecDNAs exhibited a non-random chromosomal distribution, with significant enrichment observed on chromosomes 3, 6, 7, and 8 (Figure 3E). Due to their acentromeric structure, ecDNAs segregate randomly during mitosis, allowing rapid amplification and heterogeneous distribution of oncogenes across tumor cells, thus accelerating tumor evolution and drug resistance [38]. Among the identified ecDNAs, 25% (6 out of 24) carried oncogenes, including *MYC*, *ERBB2*, *MECOM*, *NDRG1*, *PVT1*, and *GSDMC* (Figure 3F). Of particular interest was the discovery of a large ecDNA containing *HIF1A* in cisplatin-resistant SKOV3 cell lines (Figure 3G). *HIF1A* is known to promote platinum resistance by regulating genes involved in DNA repair pathways, drug efflux mechanisms, and epithelial–mesenchymal transition (EMT)-associated transcription factors [39].

### 3.4. Genome-Wide Detection and Distribution Features of Small eccDNAs in HGSOC Cell Lines

**Figure 4:**
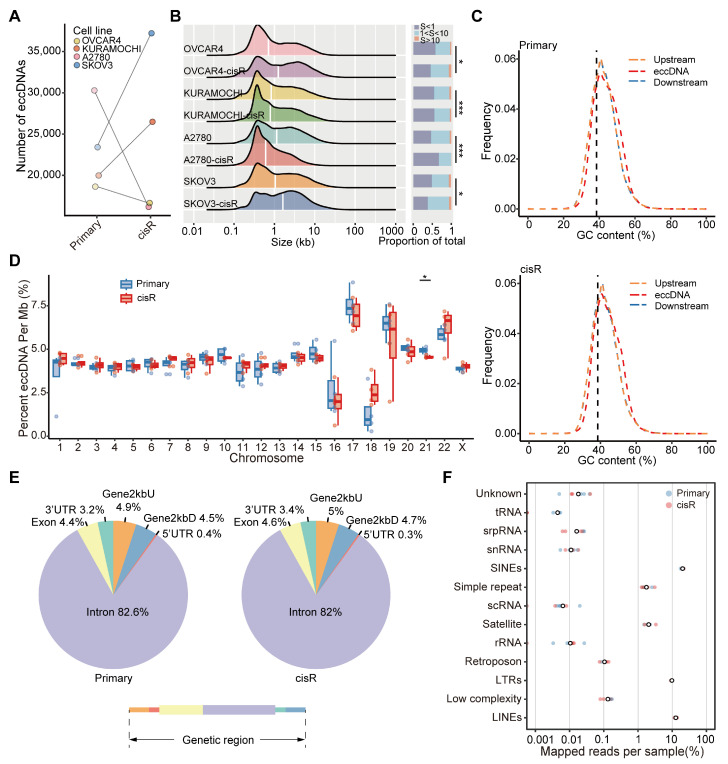
EccDNA distribution and length. (**A**) Number of eccDNAs identified in four paired HGSOC cell lines. (**B**) Length distribution of eccDNAs (**left**), with the white vertical line indicating the median. The bar plot (**right**) shows the proportion of eccDNAs in different size ranges. S, Size. (**C**) GC content of eccDNA regions and upstream/downstream regions in primary (**upper**) and cisR (**lower**) cell lines. Upstream, extends 1000 bp upwards from the eccDNA upstream coordinates; downstream, extends 1000 bp down from the eccDNA downstream coordinates. The black vertical dotted line represents the mean GC content in the genome. (**D**) Comparison of the frequency of eccDNAs per Mb of each chromosome (Paired *t*-test). (**E**) Genomic distribution of eccDNA genes in primary (**left**) and cisR (**right**) cells. The lower panel shows a simplified representation of genome distribution. (**F**) Repetitive regions from total mapped reads for each cell line-derived eccDNA. White circles represent the median. Statistical significance is indicated as follows: NS = not significant, * *p* < 0.05, *** *p* < 0.001.

Further, we investigated the differences in small eccDNAs between primary and cisR cell lines. we conducted Circle-seq to detect eccDNAs and characterized their abundance, length distribution, genomic distribution, and GC content across the four paired primary and cisR cell lines. The number of eccDNAs ranged from 18,621 to 30,205 in primary cell lines and from 16,166 to 37,222 in cisR cell lines (Figure 4A; Supplemental Table S5). Notably, the eccDNA profiles exhibited significant heterogeneity, with only a small fraction of eccDNAs shared between paired primary and cisR cell lines (Supplementary Figure S1A). Furthermore, length distribution revealed that eccDNAs less than 1000 bp were the predominant subtype in HGSOC cell lines, accounting for 50.1% in primary cell lines and 49% in cisR cell lines (Figure 4B; Supplementary Figure S1B). Next, we found that eccDNA was notably enriched in regions with high GC content compared to other genomic regions, suggesting that GC content is a common feature of eccDNAs, consistent with previous reports [40]. However, the mean GC content did not significantly differ between the primary and cisR cell lines (Figure 4C and Supplementary Figure S1C). eccDNA originated from all chromosomes, but its production frequency on chromosome 21 differed significantly between the primary and cisR cell lines (*p* = 0.0105) (Figure 4D). The distribution of eccDNA per megabase (Mb) across chromosomes correlated positively with the density of protein-coding genes per Mb (Supplementary Figure S1D, ρ ≥ 0.48) and non-coding RNA (ncRNA) per Mb (Supplementary Figure S1E, ρ ≥ 0.55). Finally, we examined the genomic origins of eccDNA by mapping it to different genomic elements. eccDNAs were predominantly enriched in introns and repetitive elements, including long interspersed elements (LINEs), short interspersed elements (SINEs), and long terminal repeats (LTRs) (Figure 4E,F).

### 3.5. Differentially Expressed Gene Profiles and Functional Analysis in Primary and cisR Cell Lines

**Figure 5:**
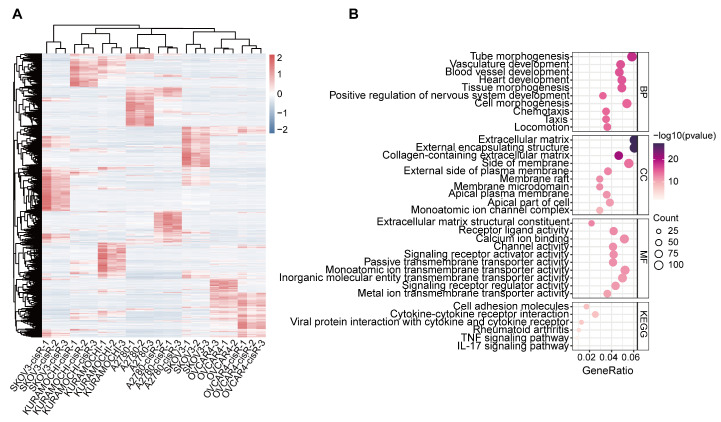
Differential expression gene between primary and cisR cell lines. (**A**) Heatmap showing the DEGs between primary and cisR cell lines. Expression values are displayed as Z scores after the values of each gene were scaled across samples. Blue, DEGs downregulated in cisR cell lines. Red, DEGs upregulated in cisR cell lines. (**B**) GO and KEGG analysis of upregulated DEGs in cisR cell lines. The top 10 terms with the lowest *p*-values were presented in the GO annotation (*p* < 0.05).

To comprehensively characterize gene expression differences associated with cisplatin resistance, we performed RNA-seq with three biological replicates per cell line to identify differentially expressed genes (DEGs) between primary and cisR cell lines. DEGs were identified using DESeq2 with stringent selection criteria (|log2 fold change| > 1; adjusted *p*-value < 0.05) (Figure 5A; Supplemental Table S6). Gene Ontology (GO) analysis revealed that upregulated DEGs in cisR cell lines were primarily associated with the extracellular matrix and synaptic membrane functions and were involved in cancer cell growth, differentiation, and various transmembrane transporter activities, including cell adhesion, cell morphogenesis, and cell projection morphogenesis (Figure 5B).

### 3.6. Differential Expression and Enrichment Analysis of eccDNA Encoding Genes

**Figure 6:**
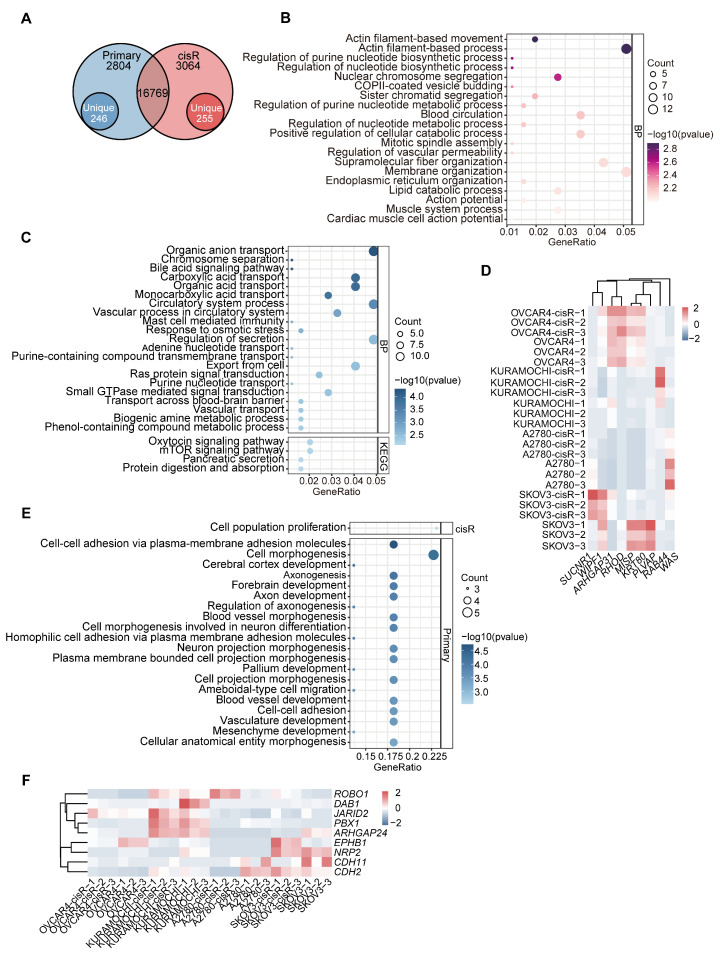
Differentially expressed eccDNA-related genes. (**A**) Uniquely differentially expressed eccDNA-related genes (DEEGs) between primary and cisR cells, respectively. (**B**,**C**) GO and KEGG analyses of upregulated DEEGs (eccDNA) in cisR (**B**) and primary (**C**) cells (*p* < 0.05). (**D**) Heatmap showing expression profiles of eccDNA-related DEEGs in primary and cisR cells. Expression values are displayed as Z scores after the values of each gene were scaled across samples. (**E**) GO analysis of upregulated DEEGs (ecDNA) in cisR and primary cells (*p* < 0.05). (**F**) Heatmap showing expression profiles of ecDNA-related DEEGs in primary and cisR cells.

A total of 22,673 genes were annotated in eccDNA, with 16,769 (73.96%) shared between primary and cisR cell lines, and 2840 (12.53%) and 3064 (13.51%) unique to primary and cisR cell lines, respectively (Figure 6A). We further refined these genes to identify 246 unique eccDNA-related genes specifically enriched in primary cell lines and 255 genes exclusively detected in cisR cell lines (Supplemental Table S7). Functional enrichment analysis using GO and KEGG revealed distinct biological processes associated with these unique eccDNA-related genes. Notably, unique eccDNA-related genes in cisR cell lines were significantly enriched in processes related to mitotic spindle assembly, cell proliferation, cytoskeletal organization, and regulation of vascular permeability (Figure 6B). Specific representative genes within these pathways included *MISP*, *WIPF1*, *RHOD*, *KRT80*, and *PLVAP* (Figure 6D). Conversely, unique eccDNA-related genes in primary cell lines were predominantly involved in biological pathways such as cellular response to signal transduction, small GTPase-mediated signaling, Ras protein signal transduction, and cell morphogenesis (Figure 6C). Furthermore, we further conducted differential expression analysis on the genes in these enrichment pathways (Figure 6D). Specific representative genes within these pathways included *MISP*, *WIPF1*, *RHOD*, *KRT80*, and *PLVAP*. To further elucidate the functional implications of ecDNA-related genes, we combined annotations derived from WGS and Circle-seq analyses. Functional enrichment analysis revealed that unique ecDNA-related genes in cisR cell lines were significantly enriched in pathways related to cell proliferation (Figure 6E). In contrast, unique ecDNA-related genes in primary cel lines were predominantly involved in processes related to cell–cell adhesion, cell morphogenesis, and blood vessel development (Figure 6E). Then, we conducted differential expression analyses of genes in the enrichment pathways of primary and cisR cells (Figure 6F). Specific representative genes within these pathways included *JARID2*, *EPHB1*, and *PBX1*.

## 4. Discussion

Extrachromosomal circular DNA has long been observed in both normal and cancerous tissues [41]. However, the characteristics of eccDNA in cisplatin-resistant ovarian cells have not yet been reported. Our study presents the first integrative analysis of eccDNA in cisplatin-resistant HGSOC cell lines, using WGS, Circle-seq, and RNA-seq across four paired primary and resistant HGSOC cell lines to explore its role in cisplatin resistance.

We first utilized WGS to characterize the differences in somatic mutations, copy number variations, and large ecDNA between primary and cisR cell lines. Consistent with previous reports, the overall genomic alterations between primary and cisR cell lines were relatively moderate, except for a significant increase in tumor mutation burden [42]. We identified a total of 24 ecDNAs (15 in cisR cell lines vs. 9 in primary cell lines). Compared to primary cell lines, cisR cell lines exhibited a higher number of ecDNAs and more complex structural variations, indicating that cisplatin treatment exacerbates genomic instability and promotes ecDNA formation. ecDNAs frequently carried oncogenes and contributed to their amplification, including *MYC*, *ERBB2*, *MECOM*, and *PVT1*. Moreover, most ecDNAs were assembled through the circularization of multiple DNA fragments rather than originating from a single DNA fragment. Importantly, we identified an ecDNA carrying *HIF1A* in the SKOV3-cisR cell line. ecDNA-mediated amplification of the *HIF1A* gene in the SKOV3-cisR cell line has been previously linked to platinum resistance by inducing miR-223 secretion from macrophages and their derived exosomes, thereby promoting therapy resistance in ovarian cancer patients [43]. Our findings align with previous studies that have identified eccDNA-mediated oncogene amplification as a mechanism of drug resistance in various cancers, including glioblastoma and hypopharyngeal squamous cell carcinoma [8,44]. However, our study is the first to report the involvement of eccDNA in cisplatin resistance in HGSOC, highlighting the potential universality of this resistance mechanism across different tumor types.

Compared to WGS, Circle-Seq is a more sensitive method for detecting extrachromosomal circular DNA and has been applied across various tissues and species [45]. Using Circle-Seq, we identified a total of 161,062 eccDNAs. In HGSOC, eccDNAs predominantly appeared as small circular DNA ranging from 0.1 kb to 1 kb and were enriched in regions with high GC content, introns, and repetitive elements such as LINEs and SINEs. Notably, eccDNAs were derived from all chromosomes, with only chromosome 21 exhibiting a significantly different production frequency between primary and cisR cell lines. Furthermore, we observed a strong positive correlation between the frequency of eccDNA formation and non-coding RNA transcription, particularly evident in cisR cell lines [11].

Furthermore, integrating RNA-seq, we identified 246 unique eccDNA-related genes in primary cell lines and 255 in cisR cell lines. Functional enrichment analysis of unique eccDNA-related genes revealed their roles in cellular proliferation, differentiation, transmembrane transport, and adhesion signaling, indicating potential mechanisms underlying cisplatin resistance. Cisplatin treatment may facilitate chemoresistance by altering cell adhesion signals and remodeling the extracellular matrix surrounding tumor cells [46]. Unique eccDNA genes specifically enriched in cisR cells participated in pathways involving mitotic spindle formation, supramolecular fiber organization, and vascular permeability regulation. Among these genes, *KRT80* was notably prominent. Elevated *KRT80* expression has been previously identified as an independent prognostic factor and potential therapeutic target in ovarian cancer [47]. *KRT80* is overexpressed in various tumors and plays a crucial role in promoting proliferation, migration, and invasion, and is associated with poor prognosis. In colorectal cancer, gastric cancer, and lung adenocarcinoma, *KRT80* has been identified as a potential biomarker [48,49,50]. High *KRT80* expression in esophageal cancer has been shown to exacerbate invasive phenotypes and contribute to chemotherapy resistance [51]. Furthermore, extracellular vesicles derived from macrophages were shown to activate *STAT3* phosphorylation by downregulating *MISP*, thereby promoting therapeutic resistance in cancer cells [52].

The identification of eccDNAs carrying *HIF1A* and *KRT80* in cisplatin-resistant HGSOC cell lines suggests potential therapeutic targets. Inhibiting *HIF1A* has been shown to sensitize resistant tumors to therapy in various cancer types, including prostate and colon cancers [53,54]. Similarly, knockdown of *KRT80* expression has been demonstrated to suppress tumor growth and chemoresistance in gastric cancer [55]. These findings underscore the potential clinical relevance of targeting *HIF1A* and *KRT80* pathways in overcoming cisplatin resistance in HGSOC.

Our findings also support the concept of treatment-induced genome chaos as a driver of resistance. HGSOC is characterized by high chromosomal instability (CIN), which can be further aggravated by cisplatin-induced DNA damage. This therapy stress may lead to the formation of polyploid giant cancer cells (PGCCs), whole-genome doubling (WGD), chromothripsis, micronuclei, and other catastrophic genomic events, thereby creating a permissive environment for eccDNA generation [56,57]. The increased eccDNA abundance observed in our cisR models may, therefore, be a downstream consequence of chemotherapy-induced genomic instability. Previous studies have demonstrated that WGD and chromothripsis play a central role in facilitating clonal evolution under therapeutic pressure, giving rise to eccDNA elements, and resistant variants [37,58]. Our results are consistent with this model, supporting a mechanistic link between CIN-driven genome chaos and eccDNA generation. Targeting or restricting CIN may help prevent the emergence of eccDNA-mediated therapeutic resistance.

In summary, we delineated the landscape and characteristics of eccDNAs in primary and cisplatin-resistant HGSOC cell lines, offering a novel perspective on the mechanisms of chemoresistance. The identification of potential targets such as *HIF1A* and *KRT80* holds promise for the development of innovative therapeutic strategies against drug-resistant ovarian cancer. Future studies should aim to validate these findings in larger cohorts and clinical specimens to further elucidate the role of eccDNA in resistance and guide targeted interventions.

## Data Availability

Raw data will be uploaded to Genome Sequence Archive (GSA) in BIG Data Center (https://ngdc.cncb.ac.cn/gsa/, accessed on 14 April 2025), Beijing Institute of Genomics (BIG), Chinese Academy of Sciences, with the accession number PRJCA034969.

## Supplementary Materials

The following supporting information can be downloaded at: https://www.mdpi.com/article/10.3390/genes16050517/s1, Figure S1: Features of eccDNA in ovarian cancer cell lines. Table S1: Histologic features of 8 ovarian cancer cell lines. Table S2: The list of somatic mutations in exons and splice sites for all cell lines. Table S3: ecDNA and other focal amplifications were detected from WGS data using the AmpliconArchitect (AA) software. Table S4: Complex structural variations were detected using AmpliconArchitect (AA) and JaBbA software. Table S5: All eccDNA detected in 8 ovarian cancer cell lines. Table S6: Differentially expressed genes between paired primary and cisR of four cell lines (|log2 fold change| > 1 and adjusted *p*-value < 0.05). Table S7: Unique eccDNA-related genes in primary and cisR cell lines.
