# Supplementary material for: Characterization of Extrachromosomal Circular DNA in Primary and Cisplatin-Resistant High-Grade Serous Ovarian Cancer
Source: Genes (Basel). 2025 Apr 29;16(5):517. doi: 10.3390/genes16050517 (PMC12111702; doi:10.3390/genes16050517)
Supplement: Supplementary file 1 [file genes-16-00517-s001.zip › Supplemental Figure.pdf]

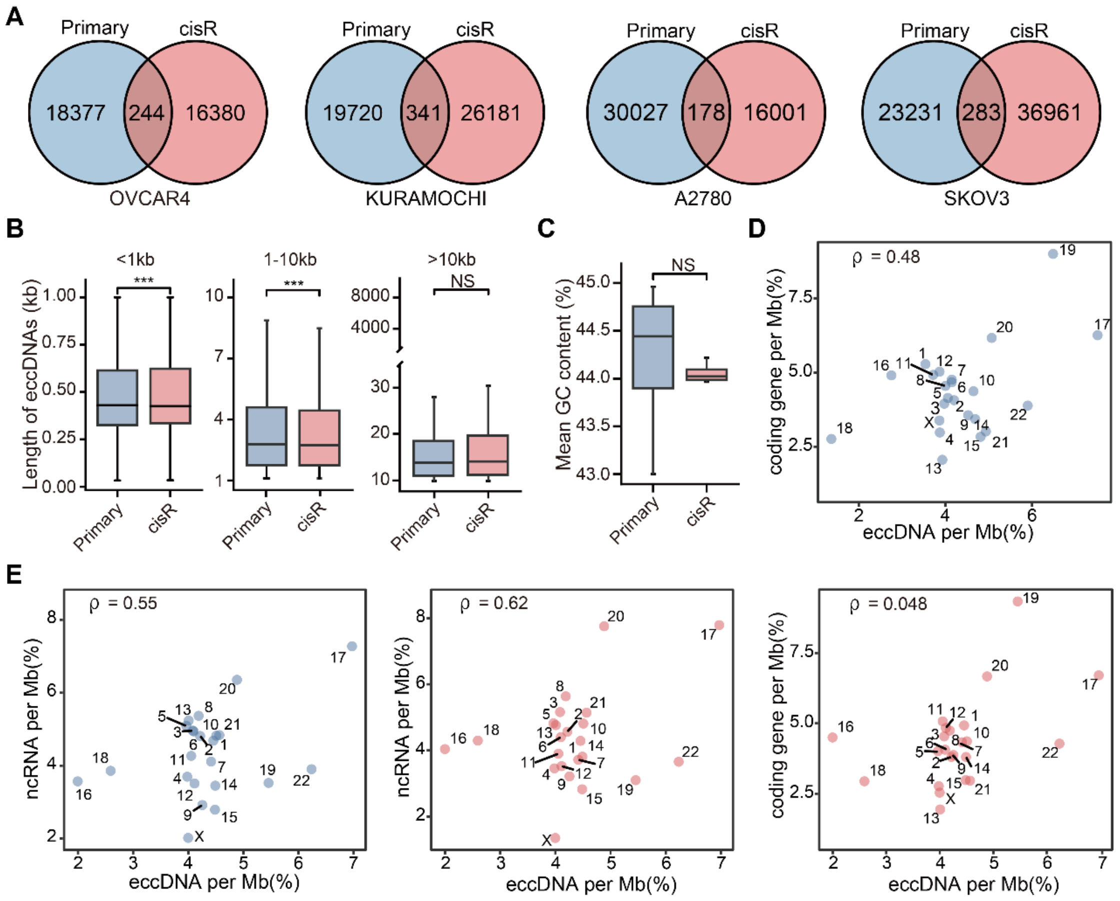

Supplemental Figure S1. Features of eccDNA in ovarian cancer cell lines.

(A) Overlap of eccDNA between each pair of matched ovarian cancer cell lines. The intersection represents eccDNA shared by primary and cisR ovarian cancer cell lines, while the non-intersecting part represents eccDNA unique to either primary or cisR cell lines. (B) Comparison of circular DNA in different length ranges between primary and cisR cells (Paired t-test, n=4). (C) Comparison of mean GC content in primary and cisR ovarian cancer cells (Paired t-test, n=4). (D) Correlation analysis between eccDNA/Mb and coding genes/Mb in the primary (upper) and cisR (lower) cell lines (n=4). Rho (ρ), Spearman rank correlation coefficient. (E) Correlation analysis between eccDNA/Mb and ncRNA/Mb in primary (left) and cisR (right) cell lines (n=4).
